# Controlled delivery of tauroursodeoxycholic acid from biodegradable microspheres slows retinal degeneration and vision loss in P23H rats

**DOI:** 10.1371/journal.pone.0177998

**Published:** 2017-05-25

**Authors:** Laura Fernández-Sánchez, Irene Bravo-Osuna, Pedro Lax, Alicia Arranz-Romera, Victoria Maneu, Sergio Esteban-Pérez, Isabel Pinilla, María del Mar Puebla-González, Rocío Herrero-Vanrell, Nicolás Cuenca

**Affiliations:** 1 Department of Physiology, Genetics and Microbiology, University of Alicante, Alicante, Spain; 2 Department of Pharmacy and Pharmaceutical Technology, Faculty of Pharmacy, Complutense University of Madrid, Madrid, Spain; 3 Department of Optics, Pharmacology and Anatomy, University of Alicante, Alicante, Spain; 4 Department of Ophthalmology, Lozano Blesa University Hospital, Zaragoza, Spain; 5 Aragon Institute for Health Research (IIS Aragon), Zaragoza, Spain; 6 Sanitary Research Institute of the San Carlos Clinical Hospital (IdISSC), Madrid, Spain; 7 Industrial Pharmacy Institute, Complutense University of Madrid, Madrid, Spain; 8 Institute Ramón Margalef, University of Alicante, Alicante, Spain; University of Florida, UNITED STATES

## Abstract

Successful drug therapies for treating ocular diseases require effective concentrations of neuroprotective compounds maintained over time at the site of action. The purpose of this work was to assess the efficacy of intravitreal controlled delivery of tauroursodeoxycholic acid (TUDCA) encapsulated in poly(D,L-lactic-co-glycolic acid) (PLGA) microspheres for the treatment of the retina in a rat model of retinitis pigmentosa. PLGA microspheres (MSs) containing TUDCA were produced by the O/W emulsion-solvent evaporation technique. Particle size and morphology were assessed by light scattering and scanning electronic microscopy, respectively. Homozygous P23H line 3 rats received a treatment of intravitreal injections of TUDCA-PLGA MSs. Retinal function was assessed by electroretinography at P30, P60, P90 and P120. The density, structure and synaptic contacts of retinal neurons were analyzed using immunofluorescence and confocal microscopy at P90 and P120. TUDCA-loaded PLGA MSs were spherical, with a smooth surface. The production yield was 78%, the MSs mean particle size was 23 μm and the drug loading resulted 12.5 ± 0.8 μg TUDCA/mg MSs. MSs were able to deliver the loaded active compound in a gradual and progressive manner over the 28-day *in vitro* release study. Scotopic electroretinografic responses showed increased ERG a- and b-wave amplitudes in TUDCA-PLGA-MSs-treated eyes as compared to those injected with unloaded PLGA particles. TUDCA-PLGA-MSs-treated eyes showed more photoreceptor rows than controls. The synaptic contacts of photoreceptors with bipolar and horizontal cells were also preserved in P23H rats treated with TUDCA-PLGA MSs. This work indicates that the slow and continuous delivery of TUDCA from PLGA-MSs has potential neuroprotective effects that could constitute a suitable therapy to prevent neurodegeneration and visual loss in retinitis pigmentosa.

## Introduction

Posterior segment eye diseases are a major cause of blindness in the world. Retinitis pigmentosa (RP) is a large and heterogeneous group of genetic disorders characterized by progressive retinal neurodegeneration. The most prevalent cause of RP is the P23H mutation in the rhodopsin-encoding gene (RHO) [1, 2], bringing on the retention of misfolded protein in the endoplasmic reticulum of rod photoreceptors [3, 4]. The degenerative mechanism of RP includes cell stress, inflammatory responses and retinal remodeling [5–10], the final common cell death pathway being apoptosis [11–15], even though non-apoptotic mechanisms may also be involved [16–18]. The decrease in the number of photoreceptors is associated with degeneration in the inner retinal layers [19–24].

Several compounds have been demonstrated efficacy in the treatment of retinal degenerative diseases. Among them, the most important component of bear bile, tauroursodeoxycholic acid (TUDCA), has been shown to display antiapoptotic effects in rodent models of retinal degeneration, including retinal light damage [25], experimental retinal detachment [26], elevated glucose-induced retinal damage [27], oxidative stress-induced retinal degeneration [28], retinitis pigmentosa [29–33], Leber congenital amaurosis [34, 35], and retinal ganglion cell (RGC) death models [36]. In these models of retinal disease, systemic administration of TUDCA significantly reduced retinal neurons death and improved retinal morphology and function. However, the doses of TUDCA given in these studies were high (500 mg/kg), which has limited to date the clinical trial of TUDCA for patients with retinal degeneration. TUDCA has also been demonstrated to be useful as an cytoprotective and antiapoptotic agent in other neurodegenerative disorders, including Huntington’s [37], Parkinson’s [38, 39] and Alzheimer’s diseases [40], in human patients with amyotrophic lateral sclerosis [41], and in models of prion disease [42].

Successful drug therapy for treating retinal degenerative diseases requires effective concentrations of neuroprotective compounds maintained over time at the site of action. However, both static and dynamic barriers effectively restrict the access of the drug to the retina after topical or systemic administration, and pose a significant challenge for ocular drug delivery in the back of the eye [43, 44]. In this context, the intraocular drug delivery systems (IODDS), capable of releasing the loaded therapeutic molecules in ocular target tissues for prolonged periods, emerge as an interesting alternative to repeated intravitreal injections of active substances in solution. The administration of IODDS not only avoids serious complications related to frequent repeated injections, but also reduce the risk of initial toxicity, due to high local drug concentrations typically occurring when they are administered in *bolus*.

Among the IODDS, the utility of biodegradable microspheres (MSs) has been previously reported [45–54]. They can be administered as suspensions using conventional needles without surgery and, as they are progressively degraded *in vivo*, no surgery is necessary at the end of the treatment. Furthermore, a personalized therapy is also possible, as the amount of MSs administered can be adjusted [50].

Biodegradable poly(D,L-lactic-co-glycolic acid) (PLGA) polymers are the materials most commonly used for the encapsulation of bioactive compounds (i.e. antiproliferatives, antiinflammatories, immunosuppressants, antibiotics and biological therapeutic agents). Approved for clinical use by the U.S. Food and Drug Administration (FDA) and the European Medicines Agency [46], they are mechanically strong, hydrophobic, biocompatible and biodegradable [44, 55]. In the treatment of vitreoretinal and macular diseases, PLGA MSs can be given by intravitreal, periocular or suprachoroidal injection [45–48, 50, 56].

The present work is focused on the preparation of TUDCA-loaded PLGA MSs for intravitreal administration and their evaluation as a potential tool for the treatment of RP. The effectiveness of TUDCA-loaded PLGA MSs in preserving retinal function and structure was evaluated by means of electroretinography (ERG) and immunofluorescence confocal microscopy on transgenic homozygous P23H line 3 rats, which undergo slow-pace degeneration of the retina. A positive valuation of the effects of TUDCA-PLGA MSs in this experimental model could lead to its potential use to treat chronic posterior segment eye diseases.

## Materials and methods

### Materials

PLGA (50:50) polymer (Resomer®503) was purchased from Boehringer Ingelheim Pharma GmbH & Co. (Ingelheim, Germany). Tauroursodeoxycholic acid (TUDCA) and polyvinyl alcohol 72000 g/mol (PVA) were obtained from Merck KGaA (Darmstadt, Germany). All organic solvents were HPLC-grade and used as received.

### Microsphere elaboration

TUDCA-loaded PLGA MSs were elaborated using an oil-in-water emulsion solvent evaporation method. Briefly, 40 mg of TUDCA were suspended in 1 mL of PLGA solution in methylene chloride (20% w/v), resulting in a TUDCA:PLGA ratio of 2:10. The prepared O-phase was emulsified with 5 mL of PVA MilliQ^®^ water solution (2% w/v) at 4,000 rpm for 1 min (Polytron^®^ RECO, Kinematica GmbH PT 10–35, Lucerna, Switzerland). The prepared emulsion was subsequently poured onto 100 mL of an aqueous PVA solution (0.1%). The system was maintained under constant stirring for 3 h to allow MSs to harden. Once formed, MSs were washed, filtered, freeze-dried and kept at -20°C and in dry conditions until used. Non-loaded PLGA MSs were prepared according to the same protocol minus the TUDCA application step.

### TUDCA quantification by LC/MS/MS

The LC/MS/MS system consisted of a Waters LC instrument with a Nova-Pak C18 column (4μm, ID 2.1 mm × 150 mm), connected to a Waters 3100 single quadrupole mass spectrometer via Empower 2 (Waters, Milford, USA). The mobile phase was composed of 33% of 15 mM ammonium acetate in water (adjusted to pH 5 with formic acid) and 67% of acetonitrile (flow rate, 0.15 mL/min). The column temperature was set to 45°C. For MS detection, the ESI source was operated in the negative ion mode. High purity nitrogen was used as the collision gas (119 L/h). Optimized MS parameters were as follows: spray voltage, 3.0 kV; capillary temperature, 180°C; capillary offset, 70 V. Meanwhile, quantitative analyses were carried out in SIR mode.

### Microsphere characterization

#### Production yield percentage (PY%)

The PY% was calculated as the percentage of MS weight divided by the total amount of PLGA and TUDCA initially used in the formulation process.

$$PY\%=\frac{weight\;of\;microspheres}{total\;amount\;of\;PLGA\;and\;TUDCA}x100$$

#### Particle size and particle size distribution

These measurements were carried out by light scattering in a Microtrac^®^ S3500 Series Particle Size Analyzer (Montgomeryville, PA, USA).

#### Morphological evaluation

The external morphology of MSs was assessed by means of scanning electron microscopy (Jeol, JSM-6335F, Tokyo, Japan) after a gold sputter-coating.

#### Encapsulation efficiency

The amount of TUDCA present in the microspheres was calculated as follows: 1 mg of MSs was placed in methylene chloride (2.5 mL). After polymer is dissolved, TUDCA was extracted with 6 mL of methanol in order to promote PLGA precipitation. After vortex mixing and centrifugation (5000 rpm for 5 min at 20°C) the methylene chloride:methanol supernatant was recuperated and filtered through 0.22 μm membranes. The TUDCA content was evaluated by LC/MS/MS as described above.

#### In vitro release studies

Duplicate samples of 5 mg TUDCA-loaded MSs were suspended in 1.5 mL of release medium, composed of PBS (pH 7.4 isotonized with NaCl), and kept at 37°C under constant agitation (Clifton Shaking Bath NE5, Nikel Electro Ldt, Avon, UK). At 24 h, at 7 days and once a week for one month, the MS suspensions were gently centrifuged (5000 rpm, 5 min, 20°C). The supernatants were recovered and replaced with an equal volume of fresh media. The TUDCA concentration in the supernatant was filtered (0.22 μm) and assayed by LC/MS/MS, as previously described.

### Animals and PLGA microspheres administration

Homozygous P23H line 3 albino rats (*n* = 17), kindly provided by Dr. M. LaVail (UCSF), were used as an animal model of RP. Age-matched Sprague-Dawley (SD) rats (Harlan, IN, USA) were used as normal controls. The animals were bred and maintained under barrier conditions of controlled temperature (23 ± 1°C), humidity (60%), and photoperiod (LD 12:12). Water and food were available *ad libitum*.

All procedures were approved by the ethic committee for animal care and use of the University of Alicante (UA-2013-07-22). All animals were housed and handled according to current regulations regarding the use of laboratory animals (NIH, ARVO and European Directive 2010/63/UE), which are intended to limit both animal suffering and the number of animals required for experimentation. All animals were anesthetized through intraperitoneal injection of a mixture of ketamine (100 mg/kg) and xylazine (4 mg/kg).

Once a month from P30 to P90 or P120 (postnatal days of age), P23H rats received intravitreal injections of TUDCA-loaded PLGA MSs in the right eyes, and blank PLGA MSs in the left eyes. Intravitreal injection of MSs was carried out under general anesthesia, as previously described [47, 48]. Briefly, the sclera was perforated with a 30-gauge hypodermic needle (1.5 mm behind the limbus) [36]. After that, MSs suspended in PBS (5% w/v) were vortexed and injected (four microliters) into the vitreous by mean of a 10-μl Hamilton syringe (Hamilton Co, Reno, NV, USA). To prevent backflow, the needle was left in place for 1 min and withdrawn slowly. Animals with retinal bleeding or lens injury were excluded from the study. Retinal function was assessed by electroretinography at P30 (n = 17), P60 (n = 17), P90 (n = 17) and P120 (n = 12). Retinal structure was characterized by immunofluorescence confocal microscopy at P90 (n = 5) and P120 (n = 9).

### ERG recordings

Animals were dark adapted overnight (12–15 h) and prepared for bilateral ERG recording under dim red light. They were anesthetized through intraperitoneal injection of a mixture of ketamine (100 mg/kg) and xylazine (4 mg/kg), and maintained at 38°C by a homeothermic heated pad. A drop of tropicamide 1% (Alcon Cusí, Barcelona, Spain) was used to dilate the pupils. Topical application of Viscotears carbomer 0.2% (Novartis, Barcelona) prevented dehydration and allowed electrical contact with the DTL electrodes (Sauquoit Industries; Scranton, PA, USA). A reference electrode, consisted of a 25-gauge platinum needle, was introduced under the scalp between the eyes, and a gold ground electrode was placed in the mouth. All experiments were performed on a Faraday cage in absolute darkness. Light stimuli were generated by a Ganzfeld stimulator and presented for 10 ms at 9 different increasing luminances (-5.2 to 0 log cd·s/m^2^). For each light presentation, three to ten successive recordings were averaged. The time between successive light flashes was 10 s for dim flashes and 20 s for the highest luminances. The recorded signals were band-pass filtered (1–1000 Hz, without notch filtering) and amplified using a DAM50 data acquisition board (World Precision Instruments, Aston, UK). Data acquisition (4 kHz) and stimulus presentation were performed by a PowerLab system (ADInstruments, Oxfordshire, UK).

### Retinal histology

#### Retinal sections

Animals were sacrificed between 10:00 a.m. and 12:00 p.m. by intraperitoneal injection of pentobarbital sodium. The dorsal margin of the limbus was marked with a stitch. The enucleated eyes were fixed in 4% paraformaldehyde during 1 h at room temperature, washed in 0.1 M PB at a pH of 7.4 (PB) and cryoprotected in 15, 20 and 30% sucrose. After removing the cornea, lens and vitreous body, the retinas were embedded in OCT and frozen in liquid N_2_. Retinal sections (fourteen-μm-thick) were prepared at -25°C by a cryostat (Leica CM 1900; Leica Microsystems, Wetzlar, Germany), mounted on adhesive slides (Superfrost Plus, Menzel GmbH & Co KG, Braunschweig, Germany) and air-dried. Before further use, retinal sections were thawed, washed in PB for three times, and placed for 1 h in blocking solution (10% normal donkey serum in PB plus 0.5% Triton X-100).

#### Retinal immunofluorescence

For an objective comparison, PLGA-TUDCA-treated and control retinas (Non-loaded PLGA MSs) were processed in parallel. The primary antibodies used (summarized in Table 1) have been characterized previously. Retinal sections were processed for single or double immunostaining with combinations of antibodies at different dilutions (Table 1) in PB with 0.5% Triton X-100 overnight at room temperature. Subsequently, anti-guinea pig IgG (Alexa Fluor 643 Conjugate), anti-rabbit IgG (Alexa Fluor 488 Conjugate) and/or donkey anti-mouse IgG (Alexa Fluor 555 onjugate) secondary antibodies (Molecular Probes, Eugene, OR, USA) were applied at 1:100 dilution for 1 h. The nuclear marker TO-PRO-3 iodide (Molecular Probes) was also added at a 1:1000 dilution. The tissue samples were washed in PB, mounted in Citifluor (Citifluor Ltd, London, UK), coverslipped and analyzed using confocal laser scanning fluorescence microscopy (Leica TCS SP2; Leica Microsystems). Negative controls included omission of the primary or secondary antibody. Confocal images were processed using Adobe Photoshop 10 software (Adobe Systems Incorporated, San José, CA, USA).

**Table 1:** Primary antibodies.

Molecular marker	Antibody (reference)	Source	Working dilution
Bassoon	Mouse monoclonal [57]	Enzo Life Sciences (ADI-VAM- PS003)	1:1000
Calbindin D-28K	Rabbit polyclonal [58]	Swant (CB-38a)	1:500
Cone arrestin	Rabbit polyclonal [59]	Chemicon (AB15282)	1:500
Protein kinase C, α isoform (α-PKC)	Rabbit polyclonal [60]	Santa Cruz Biotechnology (sc-10800)	1:100
C-terminal Binding Protein-2 (CtBP2)	Mouse monoclonal. Clone: 16/CtBP2 [61]	BD transduction (612044)	1:1000
Vesicular Glutamate Transporter 1 (VGLUT1)	Guinea Pig polyclonal [62]	Chemicon (AB5905)	1:1000

#### Retinal layer thickness

The outer nuclear layer (ONL) thickness of both TUDCA-PLGA-MSs-treated and control retinas were measured in at least 2 sections of each retina containing the optic nerve and the temporal and nasal *ora serratas*. To do this, retinal sections were counterstained with Mayer’s hematoxylin solution (#254766 Panreac, Barcelona, Spain) and eosin yellowish (#251299 Panreac). The number of photoreceptor rows was counted at 0, 1, 2, 3 and 4 mm from the optic disc to each *ora serrata*.

### Statistical analyses

A two-way analysis of variance [59] was performed to assess the effects of TUDCA-loaded PLGA MSs (TUDCA-PLGA MSs vs. blank PLGA MSs) and age (P30 to P120), as well as the interactions between them. Post-hoc pairwise comparisons were performed using Bonferroni’s test when the level of significance was 0.05 or less. Normality and homogeneity of variances were tested for the previously defined variables. Statistical analyses were conducted using the software SPSS 22.0 (SPSS, Chicago, IL, USA). Data are reported as mean ± SEM. *P* values less than 0.05 were regarded as statistically significant. All measurements were made independently by two trained observers, by using double-blind methodology.

## Results

### TUDCA-loaded PLGA MSs elaboration and characterization

**Fig 1:**
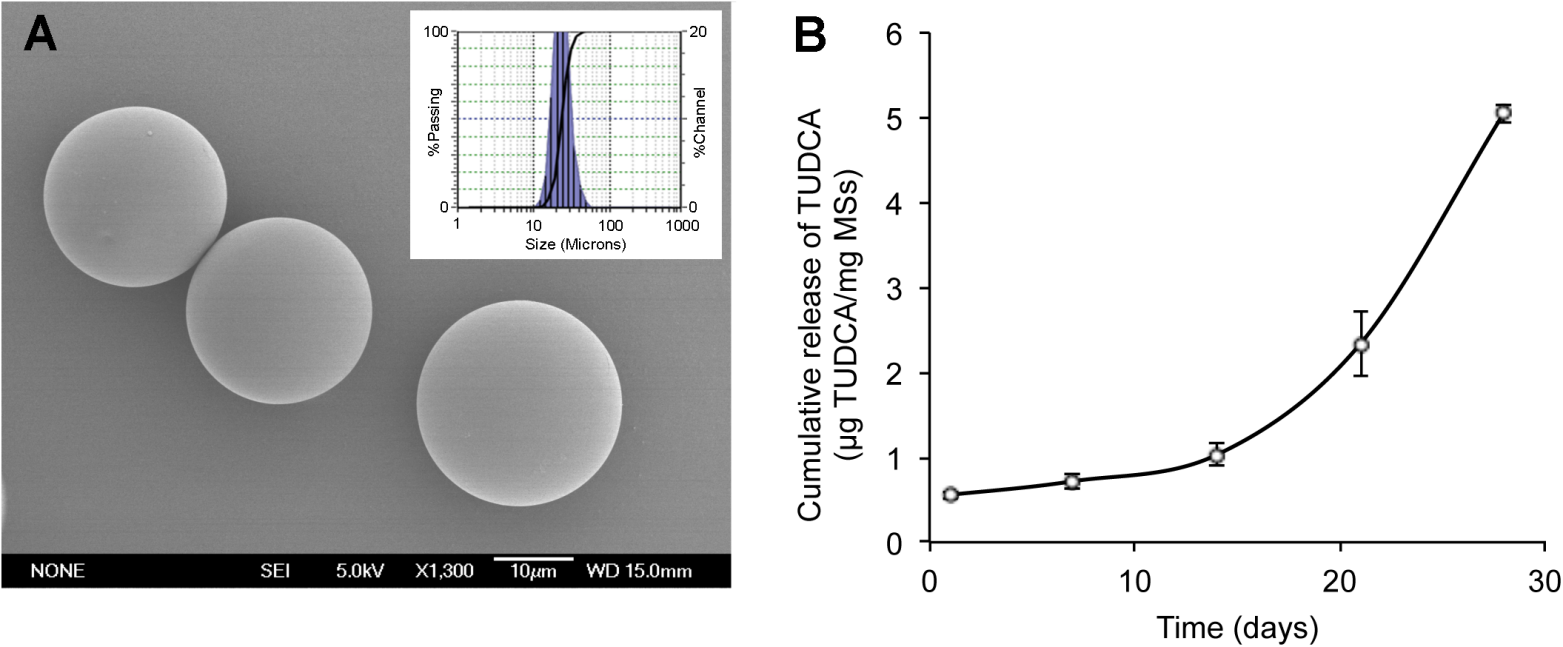
Microsphere characterization. (A) Scanning electron microscopy picture of TUDCA-loaded microspheres. Insets: particle size distribution. (B) Cumulative *in vitro* release of TUDCA (μg/mg MSs) over 28 days from TUDCA-loaded PLGA MSs in PBS (pH 7.4).

The production yield obtained using this microencapsulation method was 78.2 ± 2.1%, with the selected population of MSs in the 20–40 μm range and a mean particle size of 22.89 ± 0.04 μm. Scanning electron microscopy (SEM) pictures revealed spherical MSs with a smooth surface (Fig 1A). The encapsulation efficiency of TUDCA was 7.5 ± 0.5% (12.5 ± 0.8 μg TUDCA/mg MSs).

The amount of TUDCA released *in vitro* in the first 24 h (burst) represented 4.45 ± 0.62% (0.55 ± 0.04 μg TUDCA/mg MSs) of the encapsulated drug. After this low initial delivery, a sustained delivery was observed, with a release rate of 0.0368 μg TUDCA/mg MSs/day from day 1 to day 14, increasing to 0.2873 μg TUDCA/mg MSs/day from day 14 to day 28 (Fig 1B). A total amount of 5.05 ± 0.11 μg TUDCA/mg MSs (40.5 ± 3.5% of the total drug loading) was released at the end of the study (day 28).

### TUDCA-loaded PLGA MSs preserve retinal function

**Fig 2:**
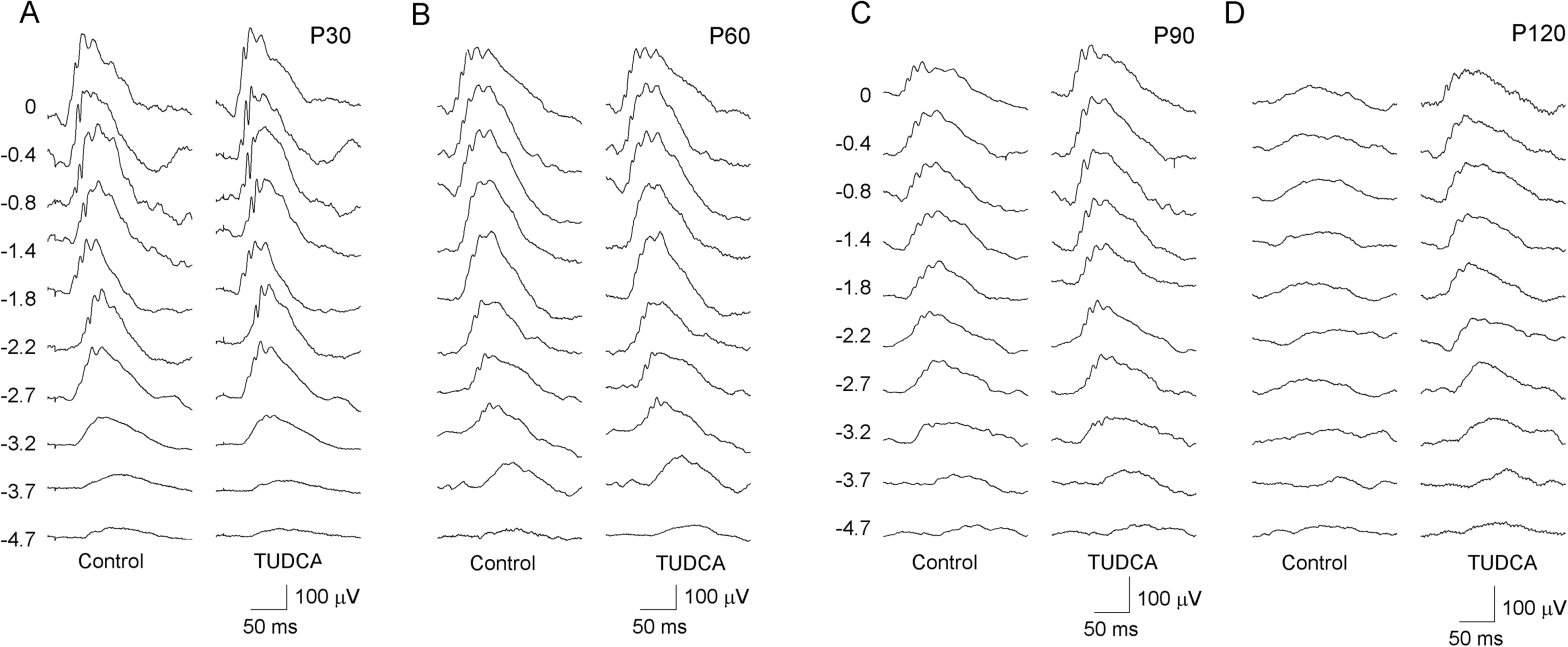
Electroretinographic responses in vehicle- and TUDCA-PLGA-MSs-treated eyes. Representative scotopic ERG traces from postnatal days P30 (A), P60 (B), P90 (C) and P120 (D) P23H rats treated with unloaded PLGA microspheres (*left*, *control*) or microspheres containing TUDCA (*right*, *TUDCA*). Units to the left of the panels indicate the flash luminance in log cd·s/m^2^. Note that ERG responses reached higher values in eyes treated with TUDCA-PLGA MSs, as compared to vehicle-treated eyes.

**Fig 3:**
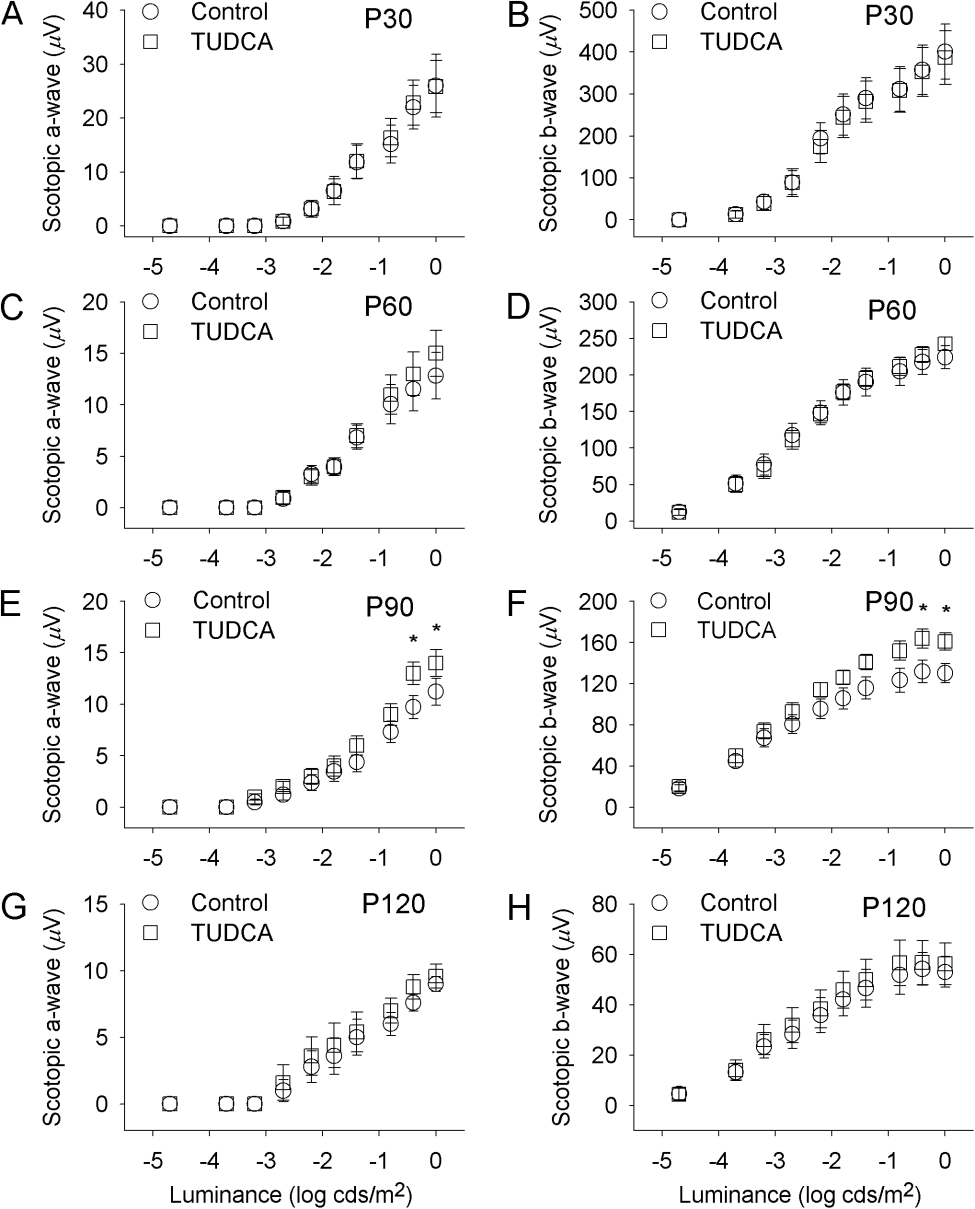
Effect of TUDCA-loaded PLGA microspheres on the ERG stimulus-response functions. Stimulus-response curves for mixed scotopic a-waves (A, C, E, G) and b-waves (B, D, F, H) from P30 (A, B), P60 (C, D), P90 (E, F) and P120 (G, H) P23H rats treated with TUDCA-PLGA MSs (*squares*) or vehicle (*circles*). ERG a- and b-waves obtained in P23H rats treated with TUDCA-PLGA MSs reached higher values than those recorded in vehicle-treated animals. *p < 0.05; ANOVA, *Bonferroni’s* test. n = 17 for P30, P60, P90 and n = 12 for P120.

To assess the effect of TUDCA-loaded PLGA MSs on the responsiveness of P23H rat retinas, scotopic ERG responses were obtained from animals treated with TUDCA-PLGA MSs (right eye) and unloaded MSs (left eye). As exampled in Fig 2, ERG responses were less deteriorated in the eyes injected with TUDCA-PLGA MSs, as compared to the controls. TUDCA-PLGA-MSs-injected eyes showed higher a- and b-wave amplitudes than that registered in blank-PLGA-MSs-injected contralateral eyes from P60, with statistically significant differences at P90 (ANOVA, *Bonferroni’s* test, *p* < 0.05 for a-waves; *p* < 0.001 for b-waves; *n* = 17) (Fig 3). Maximal differences were found at the maximum amplitudes in both a- and b-waves. At P90, the maximum scotopic amplitudes obtained for a- and b-waves were 28% and 22% higher, respectively, in TUDCA-PLGA-MSs-treated eyes, as compared to values recorded in the control eyes (ANOVA, *Bonferroni’s* test, *p* < 0.05 in both cases; *n* = 17) (Fig 3E and 3F).

### TUDCA-loaded PLGA MSs slow down photoreceptor degeneration

**Fig 4:**
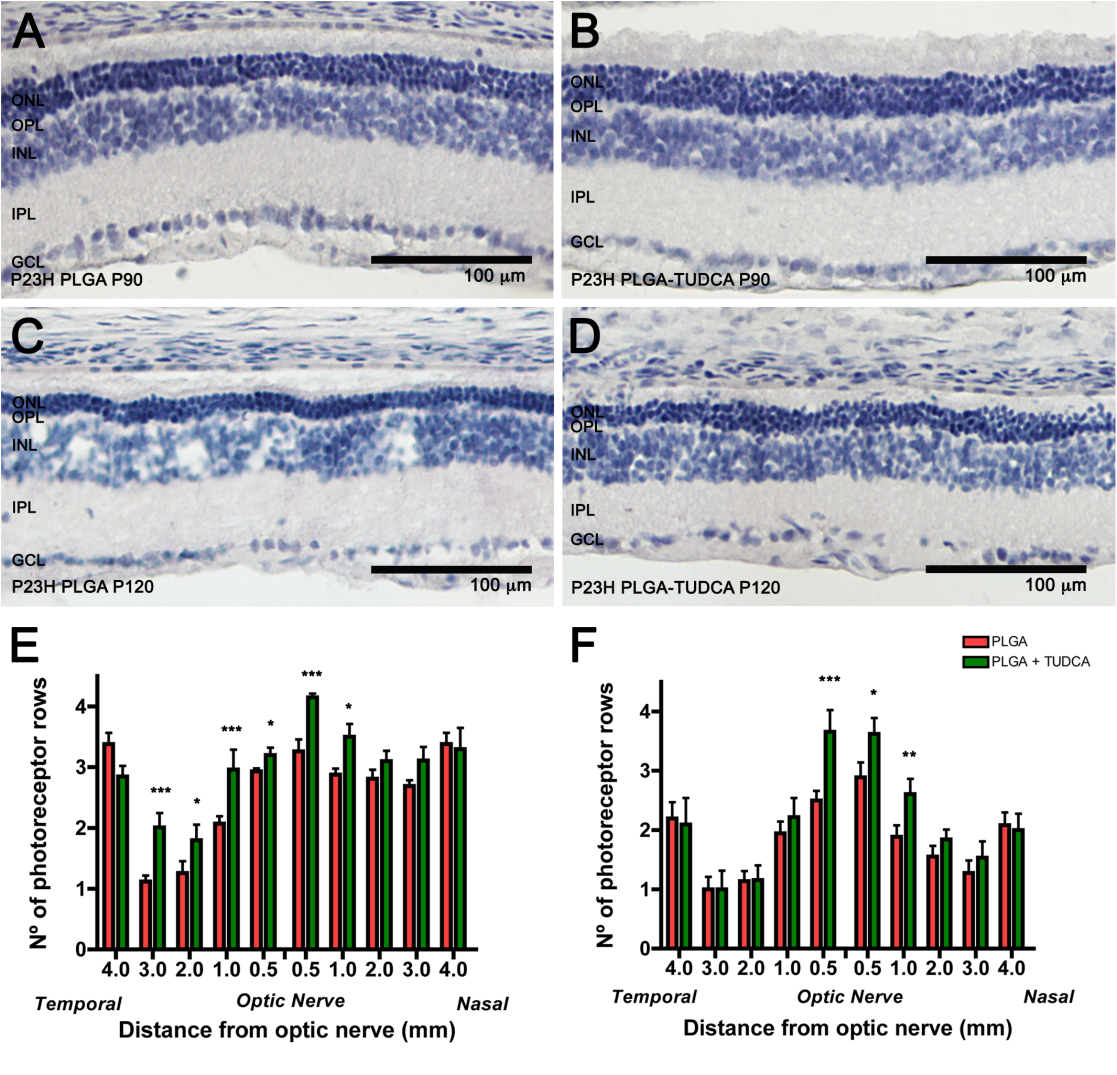
Number of rows of photoreceptor nuclei in eyes treated with vehicle or TUDCA-PLGA MSs. (A-D) Retinal sections from P23H rats at P90 (A, B) and P120 (C, D), administered unloaded PLGA MSs (A, C) or TUDCA-loaded PLGA MSs (B, D), counterstained with hematoxylin and eosin. (E, F) Mean number of photoreceptor rows along central sections of the retina in vehicle- or TUDCA-PLGA-MSs-treated animals (n = 5 and n = 9, respectively). *p < 0.05, **p < 0.01; ANOVA, *Bonferroni’s* test. ONL: outer nuclear layer, INL: inner nuclear layer, OPL: outer plexiform layer, IPL: inner plexiform layer, GCL: ganglion cell layer. Scale bar, 100 μm.

To evaluate the protective effect of the controlled delivery of TUDCA on photoreceptor cells, we measured the number of photoreceptor rows at P90 and P120, using counterstaining with hematoxylin and eosin. Fig 4 shows retinal sections of P23H rats treated with unloaded MSs (Fig 4A and 4C) and TUDCA-loaded MSs (Fig 4B and 4D). Few rows of photoreceptor cells were found in the ONL of P23H control rats at P90 (Fig 4A) and P120 (Fig 4C), as compared to the rows observed in the retina of age-matched TUDCA-PLGA-MSs-treated P23H animals (Fig 4B and 4D, respectively). Because degeneration was not homogeneous throughout the retina of control P23H rats, we assessed the effects of TUDCA-PLGA MSs in different zones of the retina, from the nasal to the temporal margins. We found that the number of photoreceptor rows was significantly greater in TUDCA-PLGA-MSs-treated than in control P23H rats at both P90 and P120 in central areas of the retina (Fig 4E and 4F, respectively; ANOVA, *Bonferroni’s* test, *p* < 0.01 for P90, *n* = 5; *p* < 0.05 for P120, *n* = 9). TUDCA-PLGA MSs showed higher neuroprotective results at P90 than at P120. In both cases, TUDCA-PLGA-MSs neuroprotective effects were maximal at the optic nerve level in the central retina, where control P23H rats showed two to three rows of photoreceptor nuclei (2.7 ± 0.2; Fig 4E), as opposed to TUDCA-PLGA-MSs-treated animals, which showed three to four remaining rows of photoreceptors (3.5 ± 0.2; Fig 4E).

### TUDCA-PLGA MSs treatment preserves photoreceptor structure

**Fig 5:**
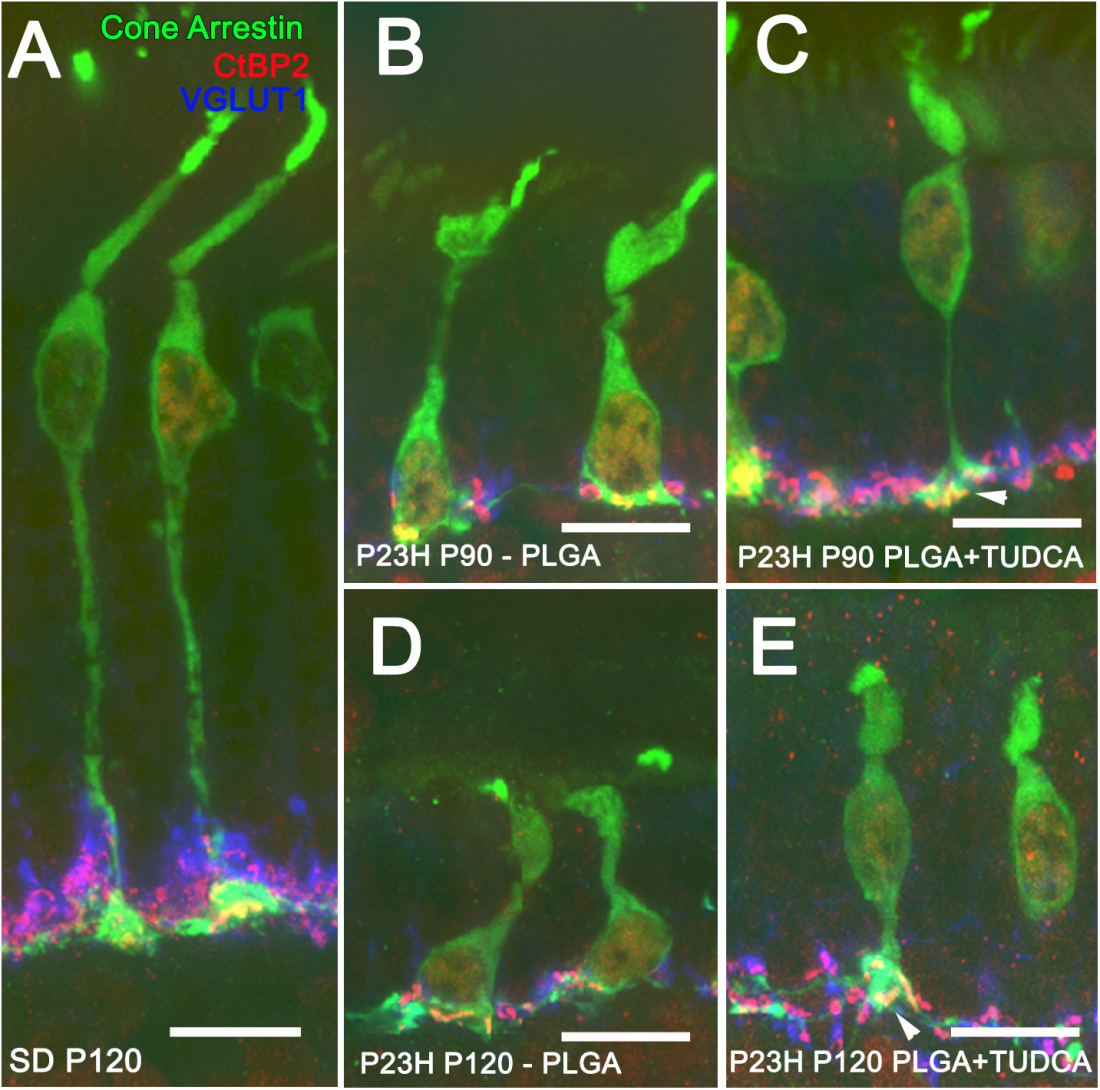
Photoreceptor morphology in vehicle- and TUDCA-PLGA-MSs-treated eyes. Triple immunolabeling for cone arrestin (in green), VGLUT1 (in blue) and CtBP2 (in red) of retinal vertical sections from a P120 normal rat (Sprague Dawley, SD) (A) and P23H rats at P90 (B, C) and P120 (D, E), treated with unloaded PLGA MSs (B, D) or TUDCA-loaded PLGA MSs (C, E). Note that the typical cone pedicles (in green) containing synaptic vesicles (in blue) surrounding synaptic ribbons (in red) were less deteriorated in TUDCA-PLGA-MSs-treated P23H rats than in vehicle-treated animals. Scale bar, 10 μm.

In order to assess whether TUDCA-PLGA MSs were able to preserve the structure of photoreceptor cells and the structure of presynaptic photoreceptor axon terminals, we analyzed the staining pattern of antibodies against cone arrestin [59], a specific marker for cones, the vesicular glutamate transporter VGLUT1 [62, 63], which contributes to the synaptic transmission between photoreceptor and bipolar cell terminals, and the C-terminal binding protein-2 (CtBP2) [61, 64], identical to the B domain of RIBEYE, a component of the presynaptic ribbons of photoreceptor cells. Fig 5A shows retinal section of a P120 wild-type Sprague-Dawley rat in which we can appreciate normal cone photoreceptors, with long axons, well-defined outer segments and typical pedicles containing numerous synaptic vesicles (in blue) that surround well-structured synaptic ribbons (in red).

In P23H rats treated with unloaded particles, retinal cone cells underwent dramatic changes with age (Fig 5B and 5D), in agreement with previously described in this animal model [29, 65]. At P90, they showed small size, short and swollen outer segments, and pedicles that directly emerged from their cell bodies (Fig 5B). At P120, the characteristic morphology of cone photoreceptors was completely lost and their cellular components were virtually unrecognizable (Fig 5D). In contrast, pedicles, axons, inner and outer segments, and the characteristic cone cell shape were less deteriorated in TUDCA-PLGA-MSs-treated animals (Fig 5C and 5E). Triple labeling using CtBP2, VGLUT1 and cone arrestin shows better preserved outer plexiform layer (compare Fig 5B and 5D with Fig 5C and 5E). In treated retinas, CtBP2 spots form a continuous band compared with the disrupted CtBP2 immunolabeling in untreated retinas (Fig 5B–5E). Also, in TUDCA treated retinas CtBP2 immunostaining appear clearly located into the preserved cone pedicles (arrowhead) at P90 and P120.

### TUDCA-PLGA MSs treatment prevents the loss of synaptic contacts between photoreceptors and bipolar cells

**Fig 6:**
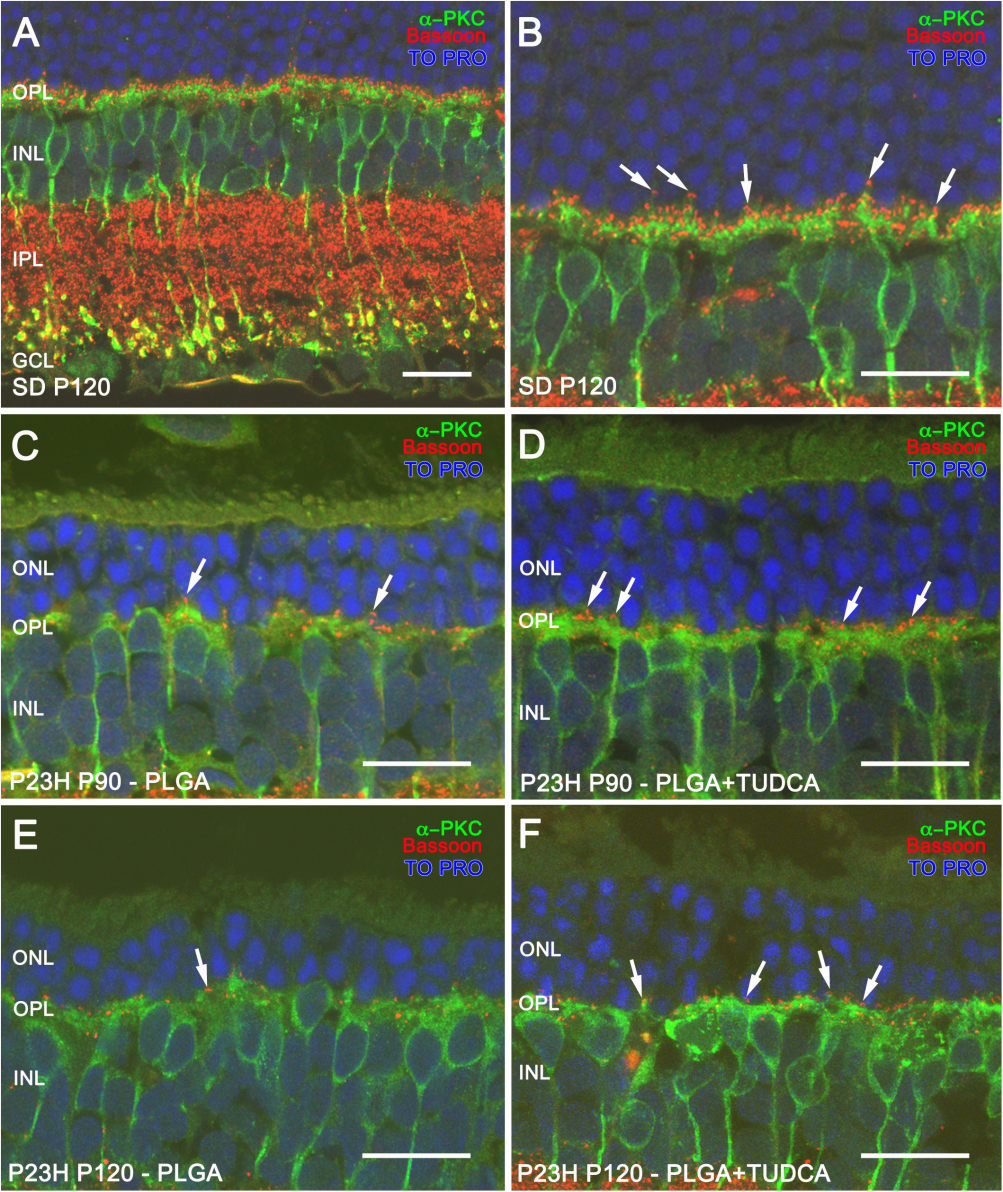
ON-rod bipolar cells and their synaptic contacts in vehicle- and TUDCA-PLGA-MSs-treated eyes. Examples of retinal vertical sections from a P120 normal rat (Sprague Dawley, SD) (A) and P23H rats at P90 (C, D) and P120 (E, F), treated with unloaded PLGA MSs (C, E) or TUDCA-loaded PLGA MSs (D, F). (B) Higher magnification of panel A. Retinal ON rod bipolar cells were stained with the α isoform of protein kinase C (α-PKC) (in green), and synaptic ribbons in photoreceptors were labeled with Bassoon (in red). Nuclei were stained with TO-PRO (in blue). Note that the density of Bassoon-positive puncta (arrows) associated with dendritic tips of bipolar cells was higher in TUDCA-PLGA-MSs-treated than in vehicle-treated P23H rat retinas. ONL: outer nuclear layer, INL: inner nuclear layer, OPL: outer plexiform layer, IPL: inner plexiform layer, GCL: ganglion cell layer. Scale bar, 20 μm.

Given that slow delivery of TUDCA treatment preserved photoreceptor cell numbers and morphology, we tested whether the protective effects of TUDCA on photoreceptor degeneration were accompanied by the preservation of synaptic contact between photoreceptor cells and second order neurons within the outer plexiform layer (OPL). To do this, retinal ON rod bipolar cells were marked with antibodies against the α isoform of protein kinase C (α-PKC) [58]. In the rat retina, rod spherules establish contacts with dendritic terminals of ON rod bipolar cells in the OPL through a large dendritic arbor (Fig 6A and 6B), and rod bipolar cell axons run into the inner plexiform layer (IPL), ending in a bulbous axon terminal (Fig 6A) [58]. In the retinas of P23H rats receiving unloaded MSs, rod bipolar cells showed a reduction of the dendritic arbor at both P90 (Fig 6C) and P120 (Fig 6E). In contrast, bipolar cell dendrites of P23H TUDCA-PLGA-MSs-treated animals were partially preserved (Fig 6D and 6F), indicating a preservation of the contacts between photoreceptors and bipolar cells.

To explore synaptic contacts between photoreceptor cells and ON rod bipolar cells in the OPL, we marked with antibodies against the protein Bassoon, a component of synaptic ribbons of both cone pedicles and rod spherules [57]. Double immunostaining for α-PKC and Bassoon evidenced the contact between the axon terminals of photoreceptor and bipolar cell dendrites (Fig 6A and 6B; arrows). In vehicle-treated P23H rat retinas labeled with antibodies against these two markers at P90 and P120, few bassoon-positive dots (Fig 6C and 6E, respectively; arrows) could be seen associated with bipolar cell dendrites labeled with α-PKC, as compared to those observed in normal SD retinas (Fig 6A and 6B). Fewer bassoon-immunoreactive spots paired with dendritic tips of bipolar cell (Fig 6D and 6F; arrows) were seen in TUDCA-PLGA-MSs-treated P23H rats as compared to SD rats, but relatively more than those observed in age-matched vehicle-treated P23H rats.

### TUDCA-PLGA MSs treatment prevents the loss of synaptic contacts between photoreceptors and horizontal cells

**Fig 7:**
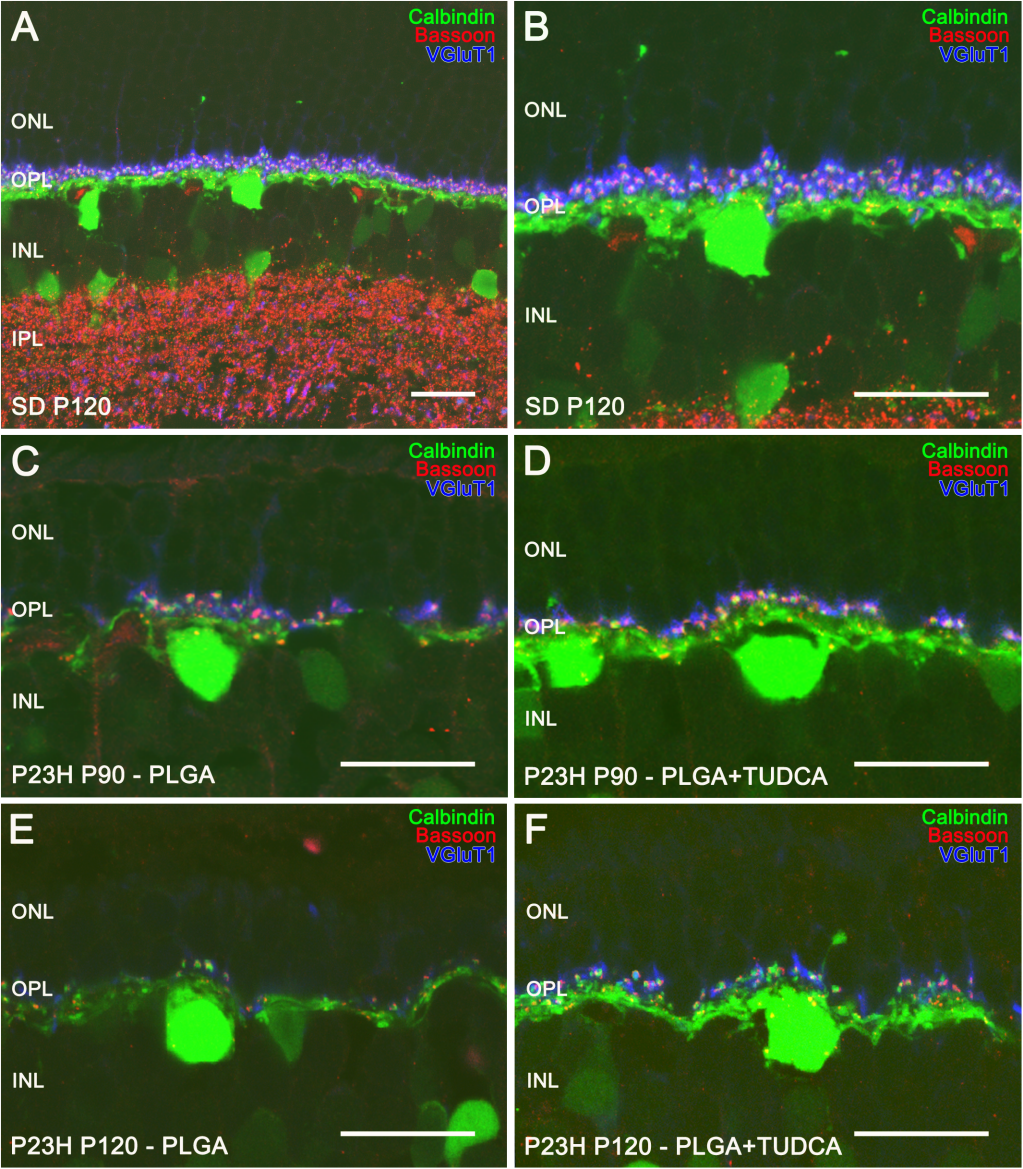
Horizontal cells and their synaptic contacts in vehicle- and TUDCA-PLGA-MSs-treated eyes. Retinal vertical sections from a P120 normal rat (Sprague Dawley, SD) (A, B) and P23H rats at P90 (C, D) and P120 (E, F), treated with unloaded PLGA MSs (C, E) or TUDCA-loaded PLGA MSs (D, F). Horizontal cells were stained for calbindin (in green), synaptic ribbons were labeled using anti-Bassoon antibodies (in red) and synaptic vesicles in the photoreceptor were stained using antibodies against VGLUT1 (in blue). Note that pairings between photoreceptor axon terminals and horizontal cell dendrites were more numerous in TUDCA-PLGA-MSs-treated retinas, as compared to those observed in vehicle-treated retinas. ONL: outer nuclear layer, INL: inner nuclear layer, OPL: outer plexiform layer. Scale bar, 20 μm.

Horizontal cells are scattered throughout the outer part of the inner nuclear layer and receive synaptic input from either rods or cones. In the rat retina, the only subtype of horizontal cell described can be marked with antibodies against calbindin [60]. Calbindin labeling in SD rats evidenced a punctate staining of dendritic terminals in the OPL, projecting from horizontal cells and contacting with the axon terminals of cones, and axonal elongations contacting with rods (Fig 7A and 7B) [60]. To better evidence synaptic contacts between photoreceptors and horizontal cells within the OPL, we used antibodies against Bassoon. Characteristic horseshoe-shaped Bassoon-positive spots were observed in P120 SD rats (Fig 7A and 7B). Photoreceptor axon terminals were also labeled with VGLUT1, a protein of glutamatergic synaptic vesicles. In SD rat retinas, VGLUT1 labeling revealed 3 or 4 rows of rod axon terminals in the OPL, each VGLUT1-labeled rod axon terminal containing a single ribbon synapse, evidenced by the Bassoon-positive spots contiguous to the tips of the horizontal cell dendrites (Fig 7A and 7B).

In P23H rats treated with unloaded PLGA microspheres, horizontal cell dendritic tips were short and scarce at both P90 (Fig 7C) and P120 (Fig 7E), concomitantly with the decrease in photoreceptor rows. Few Bassoon-immunopositive spots and reduced VGLUT1 immunostaining were found at the OPL, indicative of a decreased density of photoreceptor axon terminals (7C and 7E). Conversely, in TUDCA-PLGA-MSs-treated retinas, more horizontal cell terminals were found at both P90 (Fig 7D) and P120 (Fig 7F). Furthermore, in TUDCA-PLGA-MSs-treated rats, triple immunostaining evidenced abundant contacts between photoreceptor axon terminals and horizontal cell dendrites (Fig 7D and 7F), as compared to the fewer pairings found in vehicle-treated animals (Fig 7C and 7E) at the same ages. These results were indicative of the protective effect of TUDCA delivered from the PLGA-MSs on synapses between photoreceptor cells and horizontal cells.

## Discussion

The present study shows that PLGA microspheres containing low doses of TUDCA, when intravitreally administered, were able to slow vision loss and retinal remodeling in an animal model of retinal degeneration. The method described here allowed for the stabilization and controlled release of TUDCA encapsulated in poly(D,L-lactic-co-glycolic acid) microspheres, providing long-term intraocular delivery of the compound, and ensuring neuroprotective effects in the posterior segment of the eye.

The microencapsulation method used in this work allowed the preparation of TUDCA-loaded PLGA MSs with a good production yield and moderate encapsulation efficacy. Interestingly, little burst effect (first 24-h release) was observed, confirming the inner location of the active substance in the polymeric matrix. The biodegradation of PLGA matrixes occurs through a hydrolytic chain cleavage mechanism both on the surface and in the bulk of the system, leading to an *in vitro* release profile that typically combines slow and fast release rates [48, 66], as was observed in the *in vitro* release study.

Prepared TUDCA-loaded MSs had spherical shape and smooth surface, and a particle size suitable for intravitreal administration through conventional needles (30G-32G) after suspension in adequate vehicles [45]. Once injected, MSs act like reservoirs, progressively delivering the active substance that then has to diffuse into the vitreous gel to reach the retinal tissue [67]. Ocular tissues seem to show great tolerability and biocompatibility after the intravitreal administration of PLGA material [67–71]. Several works have evaluated the potential inflammatory reactions and damage to retinas and vision function caused by intravitreally injected PLGA microspheres and their degraded products. Although it has been described that an initial foreign body response after intravitreal injection can occurs under normal physiological conditions, similar to the ones reported for sutures, studies have confirmed that PLGA and PLA microspheres do not provoke permanent inflammation nor cell toxicity in the retinas, neither their degraded products. In any case the reaction observed disappeared 2–4 weeks after administration [67, 68].

In this work, we have used the P23H line 3 rat model of autosomal dominant RP. P23H transgenic rats mimic the clinical signs observed in patients with P23H RP [72, 73]. This animal model exhibits a primary dysfunction of rods, even though initially shows normal cone function. Apoptotic death of photoreceptors is accompanied by inner retinal neurodegenerative changes [9, 19]. Temporal course of retinal degeneration in P23H line 3 rats properly reproduces the kinetics observed in RP patients, thus giving our results further clinical significance. TUDCA-PLGA MSs were administered from P30 to P120, a phase at which P23H rats have undergone extensive retinal degeneration [29, 65].

Intravitreal administration of TUDCA-PLGA MSs in P23H rats reduced the loss of photoreceptors and preserved retinal structure, as evidenced by immunolabeling with specific antibodies. Morphological results were consistent with the high amplitudes of ERG a- and b-waves obtained in TUDCA-PLGA-MSs-treated rats, as compared with control rats. All these results are in concordance with the findings of a previous research in which we evaluated the neuroprotective effects of high doses of TUDCA (500 mg/Kg) systemically administered to P23H rats [29]. Our results also fit previous reports, which show that the systemic administration of high doses of TUDCA may preserve photoreceptor structure and function in rd mice [30–33], an animal model of RP that undergoes rapid photoreceptor degeneration [74].

Besides the protective effects of TUDCA-PLGA MSs on the density, structure and function of photoreceptor, retinas of P23H rats treated with TUDCA-loaded PLGA MSs showed preserved synaptic interactions between photoreceptor cells and secondary neurons: bipolar and horizontal cells. Both presynaptic and postsynaptic structures, as well as synaptic contacts within the OPL, were improved in P23H rats treated with TUDCA-PLGA MSs. These results suggest that the protective effect of TUDCA-PLGA MSs on the morphology and function of the retina extends to other retinal cell types in addition to photoreceptor cells. Another reasonable possibility is that photoreceptor preservation prevents the incidence of secondary neurodegenerative responses in bipolar or horizontal cells, thereby avoiding the remodeling of the entire retinal circuit [9].

In RP disease, photoreceptor degeneration normally leads to a severe remodeling of retinal pathways that limit the transmission of visual information [9]. In this study, TUDCA-loaded PLGA microspheres have proven effective in preserving photoreceptor cells from death, but also in reducing neurodegeneration at inner layers of the retina. In this context, the use of biodegradable PLGA microspheres loaded with TUDCA may be relevant in combination with other complementary therapies like the ones based on the transplantation of stem cells, anti-inflammatory or antioxidant agents and artificial chips.

The microspheres described here allowed the controlled release of TUDCA encapsulated in poly(D,L-lactic-co-glycolic acid) microspheres, providing long-term intraocular delivery of the compound and ensuring neuroprotective effects in the posterior segment of the eye. This proof of concept has demonstrated for the first time that the slow and continuous delivery of TUDCA from PLGA MSs after intravitreal administration was able to slow vision loss and retinal remodeling in an animal model for retinal degeneration. Further efforts will be made to produce micropaticulate PLGA systems able to release TUDCA in a sustained manner for longer periods of time in order to space out the intraocular injections. In conclusion, IODDS based on biodegradable MSs can provide long-term intraocular delivery of neuroprotective compounds, which is useful to reduce neurodegeneration and vision loss in retinitis pigmentosa.
